# Analysis of vertebrate vision in a 384-well imaging system

**DOI:** 10.1038/s41598-019-50372-0

**Published:** 2019-09-27

**Authors:** Robert J. Thorn, Amanda Dombroski, Kerry Eller, Tania M. Dominguez-Gonzalez, Danielle E. Clift, Peter Baek, Renee J. Seto, Elizabeth S. Kahn, Sara K. Tucker, Ruth M. Colwill, Jason K. Sello, Robbert Creton

**Affiliations:** 1 Department of Molecular Biology, Cell Biology and Biochemistry, Brown University, Providence, RI 02912 USA; 2 Department of Chemistry, Brown University, Providence, RI 02912 USA; 3 Department of Cognitive, Linguistic and Psychological Sciences, Brown University, Providence, RI 02912 USA

**Keywords:** Behavioural methods, High-throughput screening, Visual system

## Abstract

Visual impairment affects 253 million people worldwide and new approaches for prevention and treatment are urgently needed. While small molecules with potential beneficial effects can be examined in various model systems, the *in vivo* evaluation of visual function remains a challenge. The current study introduces a novel imaging system for measuring visually-guided behaviors in larval zebrafish. The imaging system is the first to image four 96-well plates with a single camera for automated measurements of activity in a 384-well format. In addition, it is the first system to project moving visual stimuli and analyze the optomotor response in the wells of a 96-well plate. We found that activity is affected by tricaine, diazepam and flumazenil. Surprisingly, diazepam treatments induce a loss of visual responses, at concentrations that do not affect activity or induce hyperactivity. Overall, our studies show that the developed imaging system is suitable for automated measurements of vertebrate vision in a high-throughput format.

## Introduction

**Figure 1:**
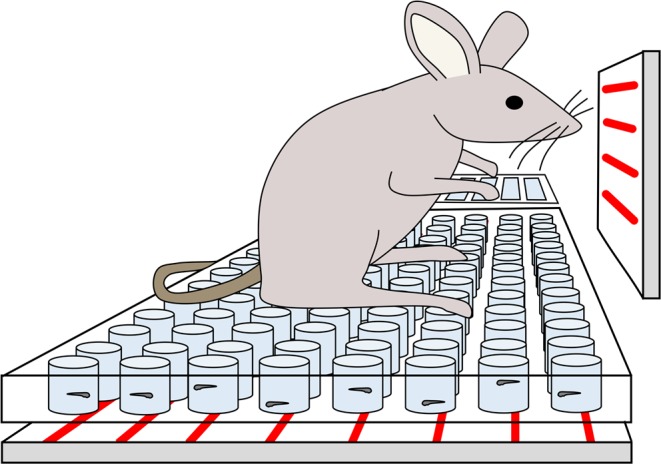
Measurement of visually-guided behaviors. Automated systems with touch screens have been developed for detailed measurements of visually-guided behaviors in rodents. However, such measurements are typically limited to a single mouse or rat per cage after months or even years of animal care. The current study presents a novel imaging system for automated analyses of 5-day-old zebrafish larvae in a set of four 96-well plates (384 wells total). This imaging system can reliably measure activity as well as responses to moving visual stimuli in a high-throughput format (microplate = 8.5 × 12.8 cm).

Visual impairment affects 253 million people worldwide: 36 million are blind and 217 million have moderate to severe vision impairment^1,2^. New approaches for prevention and treatment are urgently needed, especially for retinal disorders which have limited treatment options^3^. A persistent challenge in the development of novel treatments is how to monitor *in vivo* for functional success. A potential approach in animal model systems is the analysis of behavior, since behavioral analyses can reveal subtle functional defects, even if the visual system appears normal by morphological criteria. In addition, recent developments in automated imaging and image analysis have improved the throughput of behavioral assays in various model systems, including *Drosophila*, *C. elegans*, zebrafish and mice^4–8^. For example, automated systems with touch screens have been developed for measurements of visually-guided behaviors in rodents (Fig. 1). These measurements have provided a wealth of information on visual function and visual discrimination learning, but are typically limited to a single mouse or rat per cage^9–11^. The current study aimed to develop methodologies for automated analyses of zebrafish larvae in four 96-well plates (384 wells total) to measure vertebrate activity and vision in a high-throughput format.

## Results

### The imaging system

**Figure 2:**
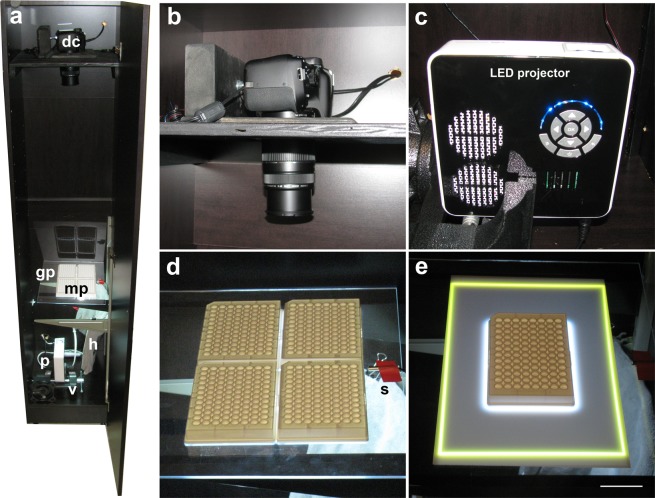
Novel imaging system for measuring behavior in 384 wells. (**a**) Overview of zebrafish imaging cabinet. (**b**) Canon T6 digital camera. (**c**) Aaxa Technologies M5 LED pico projector for presentation of visual stimuli. (**d**) Standard configuration of glass stage with four 96-well ProxiPlates. (**e**) Alternative configuration of single 96-well plate on an acrylic diffuser. A yellow rectangle is projected through the acrylic diffuser for alignment of the camera. dc = digital camera, gp = glass plate, mp = microplates, p = projector, h = heating pad, v = vise to hold projector, s = sensor of temperature control unit. Microplates are 8.5 × 12.8 cm. Scale bar in panel E = 4 cm.

**Figure 3:**
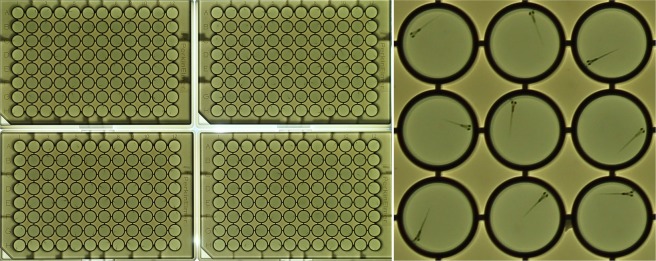
Acquired images have sufficient resolution for analyses of individual zebrafish larvae in a 384-well format. Left: Four 96-well plates. Right: cropped area of lower-left plate, well B8-D10. Inner diameter of well = 7.15 mm.

We developed a novel imaging system for automated analysis of visually guided behaviors in a 384-well format (Fig. 2). The system is built in a 180 cm tall imaging cabinet. The top shelf holds an 18-megapixel Canon T6 camera, which is connected to a laptop computer for automated image acquisition using Canon’s remote capture software. The bottom shelf holds a heater, which maintains the temperature within a 28–29 °C range and a LED pico-projector for the display of visual stimuli. The projector is connected to a second laptop with PowerPoint presentations for the control of visual stimuli. Alternatively, both the camera and projector can be connected to the same laptop. At the center of the imaging system is a glass stage, which holds four microplates. The system proved to be compatible with various microplates, including transparent 6-well and 96-well plates. The transparent plates were placed on a thin acrylic diffuser, which functions as a back-illuminated screen. Zebrafish larvae were also imaged, without the acrylic diffuser, in white ProxiPlates (PerkinElmer). The 96-well ProxiPlates have shallow wells (3.25 mm depth), providing a two-dimensional swimming area without blind spots along the well walls, even in wells along the edge of the field of view. We created moving visual stimuli in PowerPoint and projected these visual stimuli through the translucent bottom of four 96-well ProxiPlates. The high-resolution camera provided detailed views of the zebrafish larvae in each of the 384 wells (Fig. 3).

### Image analysis

**Figure 4:**
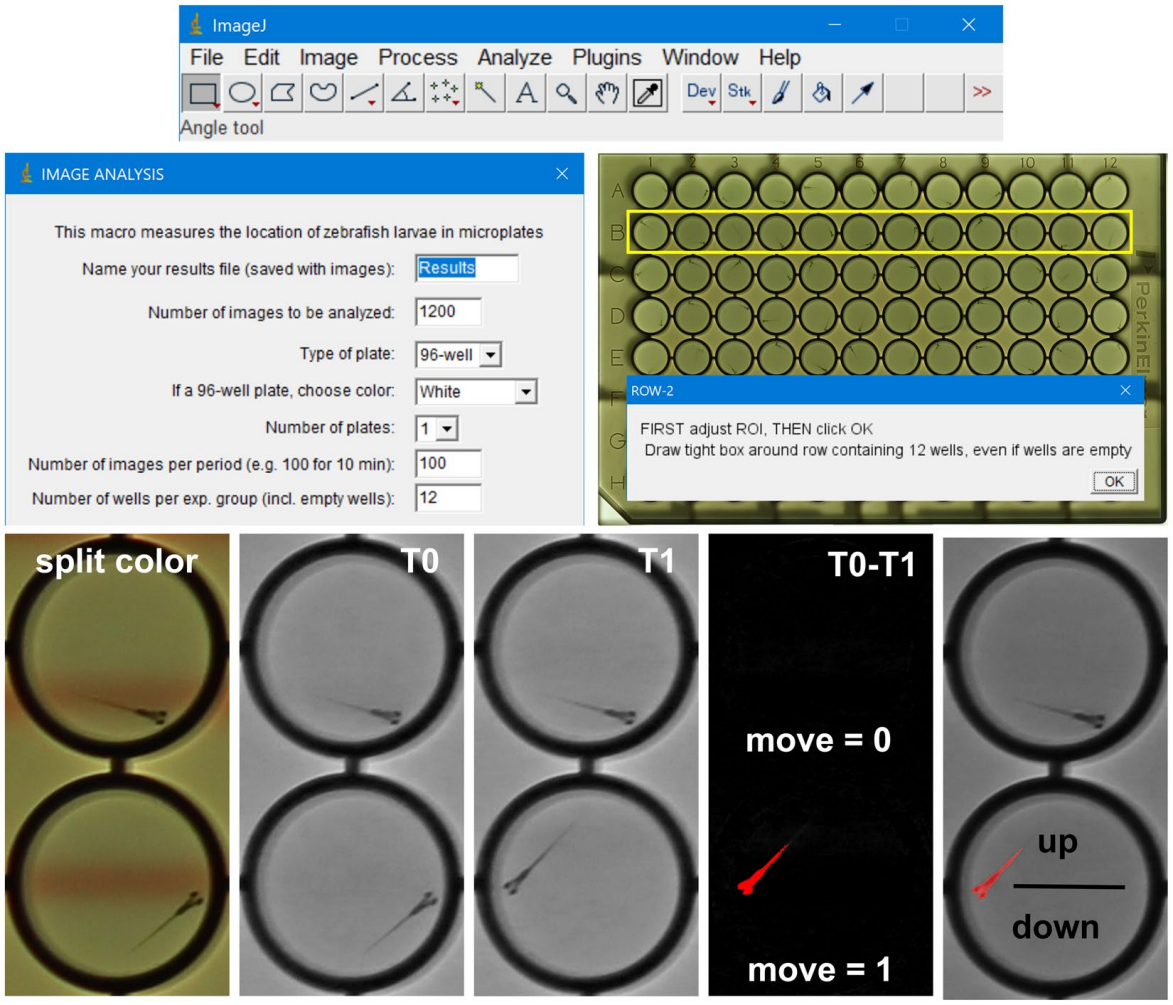
Developed software and algorithms for automated analysis of zebrafish behavior in four adjacent 96-well plates. An ImageJ dialog box gives the user various options for automated analyses of behavior. The user is asked to outline a row of wells and the software will divide this area in 12 equal regions for measurements of individual wells. The software splits the color channels and uses one of the color channels, without visual stimuli, for automated image analysis. Subsequent images are subtracted, highlighting larvae that moved. This movement is counted over time as a measure of activity. In addition, the software compares the larval centroid to the well’s centroid to determine if a larva is located ‘up’ or ‘down’ in a well. Larval location is used to examine if moving visual stimuli drive the larvae up or down a well. A single results file of a 3-hour recording contains more than 10 million data points (15 measured or calculated values × 384 wells × 1800 images).

We developed open-source software and algorithms for automated analyses of visually-guided behaviors in zebrafish larvae in a 384-well format (Fig. 4). The analysis is driven by a custom-developed ImageJ macro (Supplement 1), which splits the acquired images in three color channels, selects the channel without visual stimuli and subtracts subsequent images, highlighting larvae that move. The macro then applies a threshold and measures the area and location of the moving larvae in each of the wells. The macro repeats this process for all images in a series and then calculates larval movement and location in the upper and lower half of the well. The measured values are saved as a ‘Results’ file and are copied in a MS Excel template (Supplement 2) for data visualization. Overall, the developed imaging system and software are unique in the measurement of larval activity and responses to moving visual stimuli in a 384-well format.

### Measurements of activity

**Figure 5:**
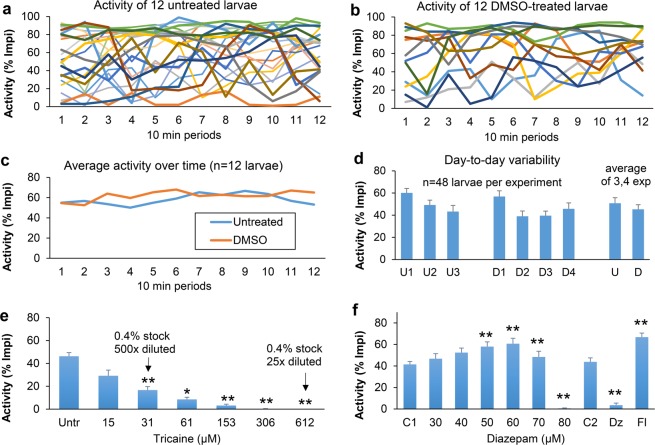
Larval activity. (**a**) Untreated 5 day-old zebrafish larvae show substantial variability in activity over time. (**b**) Similar variability is observed in larvae treated with 1 µl/ml DMSO, a solvent or vehicle that is commonly used in high-throughput screens. (**c**) Larval activity is stable over time when averaging a row of 12 larvae. (**d**) Day-to-day variability is limited when imaging four rows of 12 larvae, i.e. separate experiments carried out on different days do not display significant differences in activity (chi-squared test, n = 48 larvae per experiment). (**e**) Treatment with tricaine, a fish anesthetic. Larvae displayed reduced activity, as compared to the untreated controls, in response to a broad range of tricaine concentrations. (**f**) Treatment with diazepam (Valium), a positive allosteric modulator of the GABA receptor. Treatment with 80 µM diazepam induced a complete loss of activity. At lower diazepam concentrations (50–70 µM), larvae displayed elevated activity as compared to the DMSO-treated controls (C1). A second set of three experiments with three experimental groups per experiment confirmed the loss of activity after treatment with 80 µM diazepam and showed an increase in activity after treatment with 200 µM flumazenil, a diazepam antidote, as compared to the DMSO-treated controls (C2). Note: all measurements were made after a 1-hour acclimation period in the imaging system. In panel d-f, larval activity was averaged during a 10-minute period immediately following the 1 hour acclimation period. An asterisk or double-asterisk indicates a significant difference of a treatment group as compared to the corresponding control (*p < 0.05/n, **p < 0.01/n), using a chi-squared test with the conservative Bonferroni correction for multiple comparisons (n = 6 in panel e, n = 6 and 2 in panel f). Error bars indicate the standard error of the mean. Activity in ‘lmpi’ = larval movement per interval, using a 6 second interval.

We examined if the developed imaging system is suitable for high-throughput analyses of activity using 5-day-old zebrafish larvae. Activity levels were measured in untreated larvae and in larvae treated with 1 µl/ml DMSO, a solvent that is commonly used in high-throughput screening (Fig. 5). We found that measurements of individual larvae show substantial variability, even after a 1-hour acclimation period. When averaging activity in 12 × 10 minute periods (2 hours total), some larvae are active, some are inactive and many show substantial changes in activity over time (Fig. 5a,b). In contrast, the average activity in a row of 12 larvae is remarkably stable over time in a 2-hour recording (Fig. 5c). To examine day-to-day variability in the activity measurements, we imaged untreated 5-day-old larvae on three different days (Fig. 5d). Similarly, DMSO-treated larvae were imaged on four different days (Fig. 5d). In each experiment, larvae were imaged in four rows of 12 wells (n = 48 larvae per experiment). We found that the untreated and DMSO-treated larvae display limited day-to-day variability, i.e. activity levels at individual days are not significantly different from a pooled average of all untreated larvae (n = 144 larvae, chi-squared test). In addition, the results do not show a significant difference between the average activity in all untreated larvae (51%, n = 144) and the average activity in all DMSO-treated larvae (45%, n = 192, chi-squared test).

Activity levels were also measured in larvae treated with tricaine (MS 222), a fish anesthetic, and diazepam (Valium), an anxiolytic and sedative, which is known to affect swim speed in zebrafish larvae^12,13^.

Tricaine is typically used at a 612 µM concentration (0.4% stock, 25x diluted) for anesthesia in adult zebrafish^14^. We found that this concentration abolishes all activity in zebrafish larvae (Fig. 5e). Similarly, larvae treated with 306 µM tricaine (0.4% stock, 50x diluted) do not show any activity. Larvae treated with lower tricaine concentrations displayed activity, but at reduced levels as compared to the untreated controls. The results are based on three separate experiments, carried out on different days, with the following number of larvae in the experimental groups; n_exp1_ = 48, 24, 24, 24, 24, 24, 24 larvae; n_exp2_ = 24, 0, 12, 12, 12, 12, 12 larvae; n_exp3_ = 48, 24, 24, 24, 24, 24, 24 larvae (untreated control, 15, 31, 61, 153, 306, 612 µM tricaine respectively). Activity levels in all wells averaged 46% in untreated larvae (SEM = 3, n = 120), 29% with 15 µM tricaine (SEM = 5, n = 48), 17% with 31 µM tricaine (SEM = 3, n = 60), 9% with 61 µM tricaine (SEM = 2, n = 60), 3% with 153 µM tricaine (SEM = 9, n = 60), 0% with 306 µM tricaine (SEM = 0, n = 60) and 0% with 612 µM tricaine (SEM = 0, n = 60). The decrease in activity as compared to the untreated controls is significant in at least two out of three experiments in the 31 to 612 µM range (chi-squared test). At 31 µM, p_exp1_ = 2 × 10^−8^, p_exp2_ = 0.08, p_exp3_ = 0.001, p_median_ = 0.001. At 61 µM, p_exp1_ = 2 × 10^−12^, p_exp2_ = 0.006, p_exp3_ = 0.004, p_median_ = 0.004. At 153 µM, p_exp1_ = 6 × 10^−14^, p_exp2_ = 8 × 10^−4^, p_exp3_ = 2 × 10^−4^, p_median_ = 2 × 10^−4^. At 306 µM, p_exp1_ = 6 × 10^−14^, p_exp2_ = 8 × 10^−4^, p_exp3_ = 9 × 10^−7^, p_median_ = 9 × 10^−7^. At 612 µM, p_exp1_ = 6 × 10^−14^, p_exp2_ = 8 × 10^−4^, p_exp3_ = 9 × 10^−7^, p_median_ = 9 × 10^−7^. Based on these results, we conclude that the developed imaging system is suitable for automated analysis of zebrafish larval activity in a high-throughput format.

Diazepam is known to reduce swim speed in zebrafish larvae^12,13^. However, it is not known if diazepam can induce a complete loss of activity, which could be used as a model to study diazepam overdoses. We examined a broad concentration range and found that larvae are immotile when treated with 80 µM diazepam (Fig. 5f). The results are based on three separate experiments, carried out on different days, using 48 larvae per experiment in the DMSO controls and 24 larvae per experiment in each diazepam treatment group. Activity averaged 41% in DMSO-treated controls (SEM = 3, n = 144 larvae) and was reduced to 1% in larvae treated with 80 µM diazepam (SEM = 0.4, n = 72 larvae). This reduction was significant in all three experiments (chi-squared test, p_exp1_ = 3 × 10^−6^, p_exp2_ = 6 × 10^−8^, p_exp3_ = 3 × 10^−6^, p_median_ = 3 × 10^−6^). The diazepam-treated larvae displayed dark pigmentation, but retained their righting reflex and recover when they are transferred to a Petri dish with egg water after imaging. Surprisingly, larvae treated with 50–70 µM diazepam displayed an increase in activity as compared to the DMSO-treated controls. At 50 µM, this increase was significant in two out of three experiments (chi-squared test, p_exp1_ = 9 × 10^−4^, p_exp2_ = 9 × 10^−6^, p_exp3_ = 0.04, p_median_ = 9 × 10^−4^). At 60 µM, this increase was significant in all three experiments (chi-squared test, p_exp1_ = 2 × 10^−11^, p_exp2_ = 0.002, p_exp3_ = 5 × 10^−4^, p_median_ = 5 × 10^−4^). Similarly, at 70 µM, this increase was significant in all three experiments (chi-squared test, p_exp1_ = 1 × 10^−6^, p_exp2_ = 6 × 10^−8^, p_exp3_ = 0.002, p_median_ = 1 × 10^−6^).

In a separate set of three experiments, with 24 larvae per treatment group in each experiment, we compared larvae treated with DMSO, 80 µM diazepam, or 200 µM flumazenil, a diazepam antidote. Again, diazepam induced a strong decrease in activity. Larvae treated with 80 µM diazepam displayed 4% activity (SEM = 2, n = 72 larvae) vs. 44% activity (SEM = 4, n = 144 larvae) in the DMSO-treated controls. This decrease in activity was significant in all three experiments (chi-squared test, p_exp1_ = 7 × 10^−26^, p_exp2_ = 0.003, p_exp3_ = 3 × 10^−9^, p_median_ = 3 × 10^−9^). Flumazenil-treated larvae displayed 67% activity (SEM = 4, n = 72 larvae), which is an increase in activity as compared to the DMSO-treated controls. This increase was significant in all three experiments (chi-squared test, p_exp1_ = 0.003, p_exp2_ = 0.013, p_exp3_ = 1 × 10^−7^, p_median_ = 0.003). Based on these results, we conclude that the developed imaging system can be used to examine clinically-relevant drugs.

### Measurements of visually-guided behaviors

**Figure 6:**
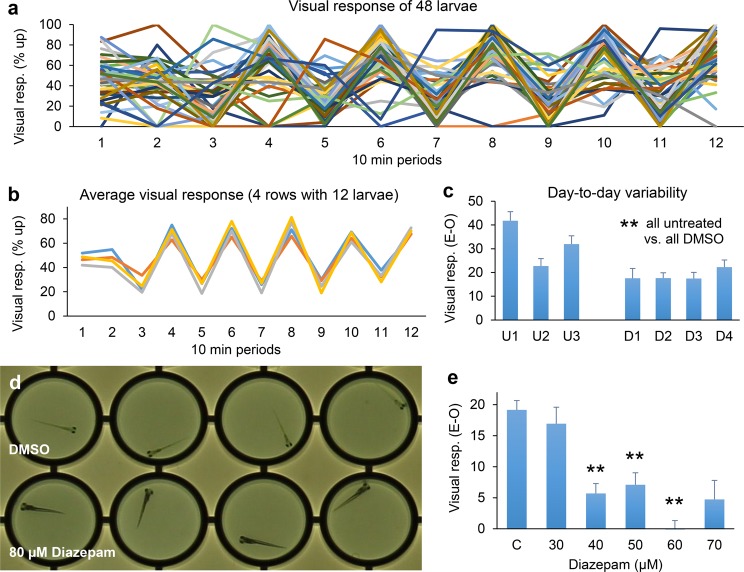
Responses to visual stimuli. (**a**) While individual zebrafish larvae display considerable variability, larvae typically move in the same direction as moving lines projected through the bottom of a white ProxiPlate. (**b**) A consistent visual response can be measured by averaging the response of 12 larvae, i.e. each line in the panel represents a row of 12 larvae. (**c**) Activity levels were significantly lower in the pooled DMSO-treated group (N = 191 larvae), as compared to the pooled untreated group (N = 143 larvae, chi-squared). Activity levels at individual days are not significantly different from a pooled average, which includes all untreated or all DMSO-treated controls (chi-squared test). (**d**) Larvae treated with 80 µM diazepam (Valium) are immotile and appear dark in the white plates. (**e**) Larvae treated with 40–60 µM diazepam display a strongly suppressed response to visual stimuli (chi-squared test, p < 0.01/5, to correct for multiple comparisons vs. the DMSO-treated control (**c**)). Error bars indicate the standard error of the mean. Visual response (E-O) = the average percentage of larvae in the upper half of the well during the Even periods (period 4, 6, 8, 10 and 12) minus the average percentage of larvae in the upper half of the well during the Odd periods (period 3, 5, 7, 9 and 11).

Prior studies have shown that zebrafish larvae display an optomotor response, i.e. larvae swim in the same direction as a series of moving lines^15,16^. We examined if these visually-guided behaviors can be detected in 96-well plates. We measured the percentage of larvae that were located in the upper half of the well (as shown in Fig. 4), while presenting lines that alternate moving up or down in 10 minute periods. Similar to the measurements of activity described above, the visual responses showed substantial variability in individual larvae, but consistent results can be obtained when averaging a row of 12 wells (Fig. 6a,b). On average, larvae were located ‘down’ in the well in periods 3, 5, 7, 9, and 11 when the visual stimuli move downwards and ‘up’ in the well in periods 4, 6, 8, 10 and 12 when the visual stimuli move upwards. The percentage of larvae in the upper half of the well during ‘Even’ periods minus the percentage of larvae in the upper half of the well during the ‘Odd’ periods (E-O) was used as a measure of the visual response (Fig. 6c). To examine day-to-day variability in the visual response, we analyzed three separate experiments using untreated larvae and four separate experiments using DMSO-treated larvae (the same files that were used to analyze day-to-day variability in activity shown in Fig. 5). The untreated groups, imaged on three different days, did not display significant differences in the visual response as compared to the pooled untreated group (n = 143 larvae). The pooled DMSO-treated group (n = 191 larvae) did display a significant reduction in the visual response, as compared to the pooled untreated group (chi-squared, p = 9 × 10^−10^). In both groups, one larva did not move sufficiently to measure a visual response. While the DMSO-treated larvae do not display gross morphological defects, it is conceivable that DMSO induces subtle morphological or functional changes in the visual system. Thus, when studying the effects of small molecules on vision, it is important to include the solvent as a control. The DMSO-treated groups imaged on four different days did not display significant differences in the visual response as compared to the pooled DMSO-treated group, suggesting that day-to-day variability will be manageable in large-scale screens. The ‘E-O’ measurements would be expected to give 50% positive and 50% negative values if larvae do not respond to visual stimuli. All untreated and DMSO-treated groups displayed a significant visual response (p < 0.01/7 or p < 0.05/7), compared to this 50–50 distribution (chi = squared test, untreated p = 2 × 10^−10^, 4 × 10^−8^, 2 × 10^−9^; DMSO-treated p = 0.004, 4 × 10^−8^, 3 × 10^−7^, 9 × 10^−7^). We conclude that the developed imaging system is suitable for high-throughput analyses of visually-guided behaviors in zebrafish larvae.

We examined the effect of diazepam on visually-guided behavior after noticing an unusual phenotype in diazepam-treated larvae. In our experiments on larval activity, diazepam-treated larvae displayed dark pigmentation in the white ProxiPlates. This dark pigmentation was most pronounced after treatment with 80 µM diazepam (Fig. 6d), but could also be observed after treatment with 40–70 µM diazepam. Prior studies have shown that defective background adaptation in zebrafish larvae is often caused by visual defects^17^, raising the question if vision is affected in diazepam-treated larvae. To answer this question, we analyzed visual responses of zebrafish larvae treated with 30–70 µM diazepam. We did not analyze vision after treatment with 80 µM diazepam, using a threshold of 5% activity for reliable measurements of vision. Moreover, larvae treated with 80 µM diazepam display morphological changes, beyond dark pigmentation, which likely affect behavior. These larvae appear swollen with an increased distance between the eyes. At the end of a 5-hour treatment, the centroid-to-centroid distance between the eyes was 350 µm (SEM = 5, n = 21 larvae) in the DMSO-treated controls vs. 472 µm (SEM = 16, n = 9 larvae) in larvae treated with 80 µM diazepam. This increased distance is significant (p = 8 × 10^−8^, chi-square test with a Bonferroni correction for multiple comparisons p < 0.001/6). We did not observe significant changes in eye distance after treatment with 30, 40, 50, 60 and 70 µM diazepam (p = 0.3, 0.3, 0.1, 0.9 and 0.1 respectively). We thus focused the analysis of visually-guided behaviors on lower concentrations of diazepam, which affect larval pigmentation, but do not suppress activity and do not lead to detectable changes in overall larval morphology. We found that diazepam induces a significant reduction in the visual response to moving red lines (Fig. 6e). Control larvae treated with the solvent DMSO displayed a 19% visual response (SEM = 2, n = 143 larvae). The visual response was 17% with 30 µM diazepam (SEM = 3, n = 71), 6% with 40 µM diazepam (SEM = 2, n = 71), 7% with 50 µM diazepam (SEM = 2, n = 64), -2% with 60 µM diazepam (SEM = 3, n = 45), and 5% with 70 µM diazepam (SEM = 3, n = 41). The decrease in vision was significant in the 40–60 µM range in at least two out of three experiments (chi-squared test). At 40 µM diazepam, p_exp1_ = 5 × 10^−4^, p_exp2_ = 0.001, p_exp3_ = 0.012, p_median_ = 0.001. At 50 µM diazepam, p_exp1_ = 9 × 10^−4^, p_exp2_ = 2 × 10^−4^, p_exp3_ = 4 × 10^−8^, p_median_ = 2 × 10^−4^. At 60 µM diazepam, p_exp1_ = 4 × 10^−4^, p_exp2_ = not available due to low motility, p_exp3_ = 1 × 10^−9^, p_median_ = 2 × 10^−4^. On average, larvae treated with 40-60 µM diazepam display normal or increased activity, indicating that activity and visual responses can be separated. Based on these results, we conclude that the developed imaging system can provide novel insights on small molecules that affect vision.

## Discussion

The current study describes the development of a novel imaging system for measuring vertebrate activity and vision in a 384-well format. This system is unique both in its capacity and capabilities.

The capacity of the developed system goes beyond the capacity of commercially-available imaging systems, such as DanioVision (Noldus) and ZebraBox (ViewPoint), which are successfully used for high-throughput analyses of zebrafish larval activity^18,19^, but are limited to a single 96-well plate per imaging system. In addition, these commercial imaging systems are not equipped for the display of moving visual stimuli and cannot measure more complex visually-guided behaviors such as the optomotor response. Prior studies have described a state-of-the-art custom-built imaging system with a projector, which can adjust visual stimuli dependent on the orientation of a zebrafish larva^15^. While this previously-described system is ideally suited for measurements of the optomotor response, it is limited to a single larva per imaging unit. The imaging system presented in the current study is also different from a prior imaging system developed in our own laboratory, which is capable of projecting visual stimuli in 6-well plates, but cannot measure visually-guided behaviors in 96-well plates^16^. In conclusion, the imaging system presented in the current study is the first to image 384 wells with a single camera and is the first to project moving visual stimuli in 96-well plates. In addition, the imaging system can be duplicated on a limited budget using widely available components, has freely available open-source software for image analysis and is easy to use. Visual stimuli, created in PowerPoint, are easily adjusted. The analysis of large imaging files is automated in ImageJ and the subsequent data analysis in MS Excel is readily modified depending on the experimental setup and needs of an investigator.

The developed imaging system was used to examine activity in 5-day-old zebrafish larvae. The results from these studies indicate that the imaging system can be used to evaluate clinically-relevant sedatives and antidotes. We found that treatments with 80 µM diazepam (Valium) induce a complete loss of activity. This loss of activity is associated with morphological changes, including a swollen phenotype with an increased distance between the eyes. Possibly, treatment with 80 µM diazepam induces morphological changes, which in turn affect larval activity. Alternatively, treatment with 80 µM diazepam may lead to a suppression of neural activity, which in turn affects larval activity, larval physiology and eventually larval morphology. Interestingly, we found that flumazenil, a diazepam antidote, induces an increase in larval activity. It is notable that flumazenil is used as an antidote to treat benzodiazepine overdoses in humans, but can lead to various complications that are in part due to its short half-life^20^. We propose that the developed imaging system may be used in high-throughput screens for novel sedatives and antidotes that are both safe and effective.

To our surprise, we found that diazepam affects vision in zebrafish larvae. We first noticed a loss of background adaptation after treatment with 80 µM diazepam, an effect that was clearly visible in the acquired images. The diazepam-treated larvae displayed dark pigmentation on a white background, which we did not see in the solvent-treated controls. Prior studies have shown that background adaptation in zebrafish larvae depends on sensing of light, a hormonal response, and a redistribution of melanosomes^17^. Thus, many blind fish have a dark color, even on a white background. We examined larvae treated with 30–70 µM diazepam and found that these larvae show normal or increased activity, with a significant reduction in their response to visual stimuli. For example, larvae treated with 60 µM diazepam displayed a complete loss of visual responses and may be considered blind. Changes in visual perception have previously been examined in rats treated with the GABAergic positive allosteric modulators chlordiazepoxide and diazepam^21^. This study not only shows that GABA receptors play a role in vision, but also highlights some of the challenges in studying vision in a mammalian model system. In the study, a group of 24 rats received more than 20 training sessions, with hundreds of trials, to perform a visual discrimination task. The rats were then injected with a drug and were tested individually in an apparatus containing a touch screen. The injection and testing was repeated in all 24 rats (a within-subject design), using various drugs at three different concentrations. These studies showed that chlordiazepoxide, but not diazepam, affected visual perception. A larger study with additional modulators of GABA receptors, using a broad range of concentrations and exposure periods, could provide a better understanding of the chlordiazepoxide specificity. Such studies would require enormous resources when carried out using a mammalian model system, but would be quite feasible in zebrafish using the developed imaging system for automated analyses of visually-guided behaviors.

Overall, our studies show that the developed imaging system is suitable for measurements of vertebrate activity and visual responses in a 384-well format. We hope that this system will be used by others for the discovery of novel small molecules with clinical relevance.

## Methods

### Zebrafish

The research project has been conducted in accordance to local and federal guidelines for ethical and humane use of animals and has been reviewed and approved by the Brown University Institutional Animal Care and Use Committee. Embryos were collected and grown to larval stages as described previously^16^. Briefly, adult wild type zebrafish (*Danio rerio*) were originally obtained from Carolina Biological and have been maintained at Brown University as a genetically diverse outbred strain. Zebrafish spawn in the morning when kept on a 14 hr light, 10 hr dark cycle in a mixed male and female population. A few tanks with adult fish will produce hundreds of embryos on a daily basis. Zebrafish embryos from 0–3 days post-fertilization (dpf) and zebrafish larvae from 3–5 dpf were grown at 28.5 °C on a 12 hour light/12 hour dark cycle in egg water, containing 60 mg/l sea salt (Instant Ocean) and 0.25 mg/l methylene blue in deionized water. The embryos and larvae were grown in 2L culture trays and were assigned randomly to different experimental groups prior to experimental manipulation or imaging. The sex of embryos and larvae cannot be determined at these early stages, since zebrafish use elusive polygenic factors for sex determination and both males and females have juvenile ovaries between 2.5 and 4 weeks of development^22^. Zebrafish larvae were imaged at 5 dpf when the larvae use nutrients that are available in their yolk sac and display a range of locomotor behaviors. The larvae are approximately 4 mm long at this time.

### Modulation of larval activity

To modulate larval activity, 5-day-old zebrafish larvae were treated with tricaine (MS-222, a fish anesthetic), diazepam (Valium®), or flumazenil (a Valium antidote). Tricaine (Syndel USA) was dissolved in deionized water as a 0.4% stock (0.4 g/100 ml, adjusted to pH 7.2 with sodium bicarbonate) and then diluted 25–1000x in egg water. The 25x dilution corresponds to a final tricaine concentration of 160 mg/L (612 µM). Diazepam (Sigma D0899) was diluted to 10–80 µM in egg water, using 10–80 mM stocks in DMSO (dimethyl sulfoxide). Flumazenil (Fisher Scientific 50-101-1113) was diluted to 100 µM using a 20 mM stock in DMSO. The corresponding DMSO concentrations were used as vehicle controls (e.g. 1 µl/ml when using 1000x stocks). Larvae were treated for 5 hours total, including an initial 1-hour incubation in a culture dish, a 1-hour period to transfer larvae to 96-well plates (1 larva per well with approximately 100 µl of treatment media), a 1-hour acclimation period in the imaging system at 28–29 °C with the projector showing a blank slide (white background) and a 2-hour imaging period (white background or visual stimuli). Unless noted otherwise, activity levels were measured in the 10-minute period immediately following the acclimation period.

### A 384-well imaging system

We developed a novel 384-well imaging system for high-throughput analyses of visually-guided behaviors. The system is based on a prior imaging cabinet^16^, with various modifications for automated analyses of behavior in a 384-well format (Fig. 2). An equipment and source list is provided in the Supplementary Information (S4). The modifications include a projector capable of displaying visual stimuli through the bottom of translucent microplates, temperature control, a glass stage, a removable acrylic diffuser and novel software for automated image analysis. The basic configuration of the cabinet and camera has been described previously^16^. Briefly, the imaging system is housed in a 180 × 40 × 40 cm cabinet. The top shelf of the cabinet holds an 18-megapixel Canon EOS Rebel T6 digital camera with an EF-S 55–250 mm f/4.0–5.6 IS zoom lens. The camera is set at ISO 100, 1/5 shutter speed, F5.0 aperture, and daylight white balance. The camera is connected to a continuous power supply (Canon ACK-E10 AC Adapter) and is controlled by a laptop computer using Canon’s Remote Capture software (EOS Utility, version 3), which is included with the camera. The software is set to interval mode to acquire high-resolution images every 6 seconds. The bottom shelf of the cabinet holds a M5 LED pico projector (Aaxa Technologies) with a 900 lumens LED light source, which is used to display visual stimuli to the larvae. The projector is held in place with a Bessey 4.5 inch bench vise and is connected to an Acer laptop for the display of PowerPoint presentations with visual stimuli. The projector has two major advantages in comparison to the previously-used LCD screen^16^: (1) the projector is brighter, which makes it possible to project visual stimuli through the bottom of translucent plates, and (2) the projector is positioned at a distance from the microplates providing space for accurate temperature control. The projector by itself raises the temperature a few degrees above ambient and the temperature is further controlled using a 15 × 12 inch heating pad (Millard) and a temperature control unit with a sensor thermostat (GeekTeches, TMC-1000) set at 28–29 °C. For overnight recordings, a humidifier (tray with a wet sponge) can be placed between the projector and heating pad. The stage of the imaging system consists of an adjustable shelf track and brackets (Rubbermaid) that hold a glass plate (Home Depot, model 91114, 11 × 14 inches). A translucent diffuser is placed on the glass stage as back-illuminated projector screen (ePlastics, 2447 white acrylic sheet, 0.060 inch thick, custom cut to 11 × 7 inch panels). This diffuser is used for imaging behavior in transparent microplates with or without visual stimuli, or imaging behavior in white 96-well ProxiPlates (PerkinElmer, 6006290) without visual stimuli. The diffuser is removed when imaging behavior in white ProxiPlates with visual stimuli, using the bottom of the white plates as a translucent back-illuminated projector screen. The ProxiPlates have shallow wells (7.15 mm diameter and 3.25 mm depth), which allows for imaging of four 96-well plates with a single high-resolution camera, while avoiding blind spots in the outer wells. In summary, the zebrafish imaging system was built with widely available components and can be duplicated on a limited budget.

### Assay for visually-guided behaviors

Zebrafish larvae were imaged for 2 hours in 96-well ProxiPlates, after a 1-hour acclimation period in the imaging system with the projector showing a blank slide with a light gray background. The larvae were first imaged for 20 minutes on a blank background and then for 100 minutes in the presence of visual stimuli. The visual stimuli consist of red lines that alternate moving downwards and upwards in 10-minute periods. The red lines have a thickness of 1 mm, are spaced apart by 7 mm, and move at a speed of 7 mm/8 seconds. The brightness of the background (RGB = 191, 191, 191) and red lines (RGB = 255, 0, 0) in the PowerPoint presentation are carefully matched to the camera settings for optimal color separation in the ImageJ macro. A PowerPoint presentation with the moving red lines is included in the Supplementary Information (Supplementary Information S3). Alternatively, larvae were imaged on a blank background without visual stimuli, or were imaged for longer periods, including the 1-hour acclimation period.

### Image analysis

We developed a novel ImageJ macro for automated analysis of behavior in a 384-well format (Fig. 3). This macro (version 26rc091018) can analyze four 96-well plates, with multiple treatment groups and changing visual stimuli over time. The software asks the user to enter information about the plates and the periods with different visual stimuli. It opens the first image, splits the color channels, and selects a channel in which the visual stimuli and background have similar intensities. The software provides an option for background subtraction in 6-well plates or uses subtractive analysis for 96-well plates. The subtractive analysis subtracts image 001–002, image 002–003 and so on, highlighting larvae that move, while hiding structures such as the well walls that do not move. The software then applies a threshold (25–255), selects the first well, measures the area with a threshold, measures the centroid of the area and logs the measurements in a ‘Results’ file. This process is automatically repeated for all wells in an image and all subsequent images in a series. When all images are analyzed the software adds a ‘Move’ column in the Results file and enters ‘1’ each time a larva moves (measured area is 20 pixels or more) or a ‘0’ when a larva does not move (measured area is smaller than 20 pixels). Similarly, the software adds an ‘Up’ column and enters ‘1’ when a larva located in the upper half of the well and a ‘0’ when a larva is located in the lower half in the well. The Results file is then imported into a MS Excel template (version 26rc091018 - for 96-well plates). This template averages values for activity and vision in all experimental groups and imaging periods and displays the results in a graph. The ImageJ macro and MS Excel templates are available in the Supplementary Information (S1 and S2) and future updates will be posted on Brown University’s central zebrafish website: https://www.brown.edu/research/projects/zebrafish/ The original results files and/or images will be made available upon request.

### Statistical analysis

For each zebrafish larva, measurements of activity and visual responses were averaged in 10-minute periods using MS Excel. The 10-minute values were then averaged in a number of larvae (e.g. n = 24 larvae using two rows of 12 wells). Differences between experimental groups and the corresponding controls were tested for significance. An analysis of normality was carried out using the Shapiro-Wilk test in IBM’s SPSS 25 software. This test revealed that both activity and visual responses do not follow a normal distribution (p = 0.001 and 0.004 respectively). Similarly, measurements of eye-to-eye distances do not follow a normal distribution (p < 0.001). Subsequent statistical analyses were carried out in MS Excel using the chi-squared test, a non-parametric test that does not require a normal distribution. Activity levels were grouped in four quartiles, i.e. 0–25%, 25–50%, 50–75% and 75–100% active. Visual responses were divided in three groups: <0%, 0–10% and >10%. In a positive response, larvae swim in the same direction as the moving visual stimuli. These larvae are located in the upper half of the well when visual stimuli move up and in the lower half of the well when the visual stimuli move down. Eye-to-eye distances were divided in two groups: 7 pixels or less and 8 pixels or more (1 pixel = 48.3 µm). Differences in behavior were considered significant when p < 0.01 or p < 0.05 with a Bonferroni correction for multiple comparisons (p < 0.01 or p < 0.05 divided by the number of comparisons). The conservative Bonferroni correction helps to avoid type I errors (false positives), which is important for the development of novel assays. Unless noted otherwise, experiments were carried out using four rows of 12 control larvae and two rows of 12 larvae per treatment group (n = 48 control larvae and n = 24 larvae per treatment group). Experiments were repeated three times, on different days. By separating experiments by at least one day, all experimental procedures including embryo collection, culture, treatment and imaging are carried out separately. Since the untreated and DMSO-treated controls showed limited day-to-day variability (Figs 5, 6), it may be possible to pool the data from separate experiments and carry out a chi-squared test on all wells. This approach has excellent statistical power and may be appropriate for large-scale screens, as long as internal controls are included in each imaging session. In the current study, we used a more conservative approach to assure independence of samples. In each of the three repeat experiments, treatment groups were compared to the corresponding controls using a chi-squared test. The p-values for all three experiments are included in the results section. The median p-value is used to show statistical significance in the graphs. Overall, this analysis is sensitive, compares treated groups to the appropriate internal controls, maintains independence of samples within a statistical test, and does not require a normal distribution.

## Supplementary information

Supplement 1

Supplement 2

Supplement 3

Supplement 4

## Data Availability

The datasets generated and analyzed in the current study are available from the corresponding author on request. The software for data analysis is included in the Supplementary Information.
