# Supplementary figures and images for: Analysis of vertebrate vision in a 384-well imaging system
Source: Sci Rep. 2019 Sep 27;9:13989. doi: 10.1038/s41598-019-50372-0 (PMC6764987; doi:10.1038/s41598-019-50372-0)

## Slide 1
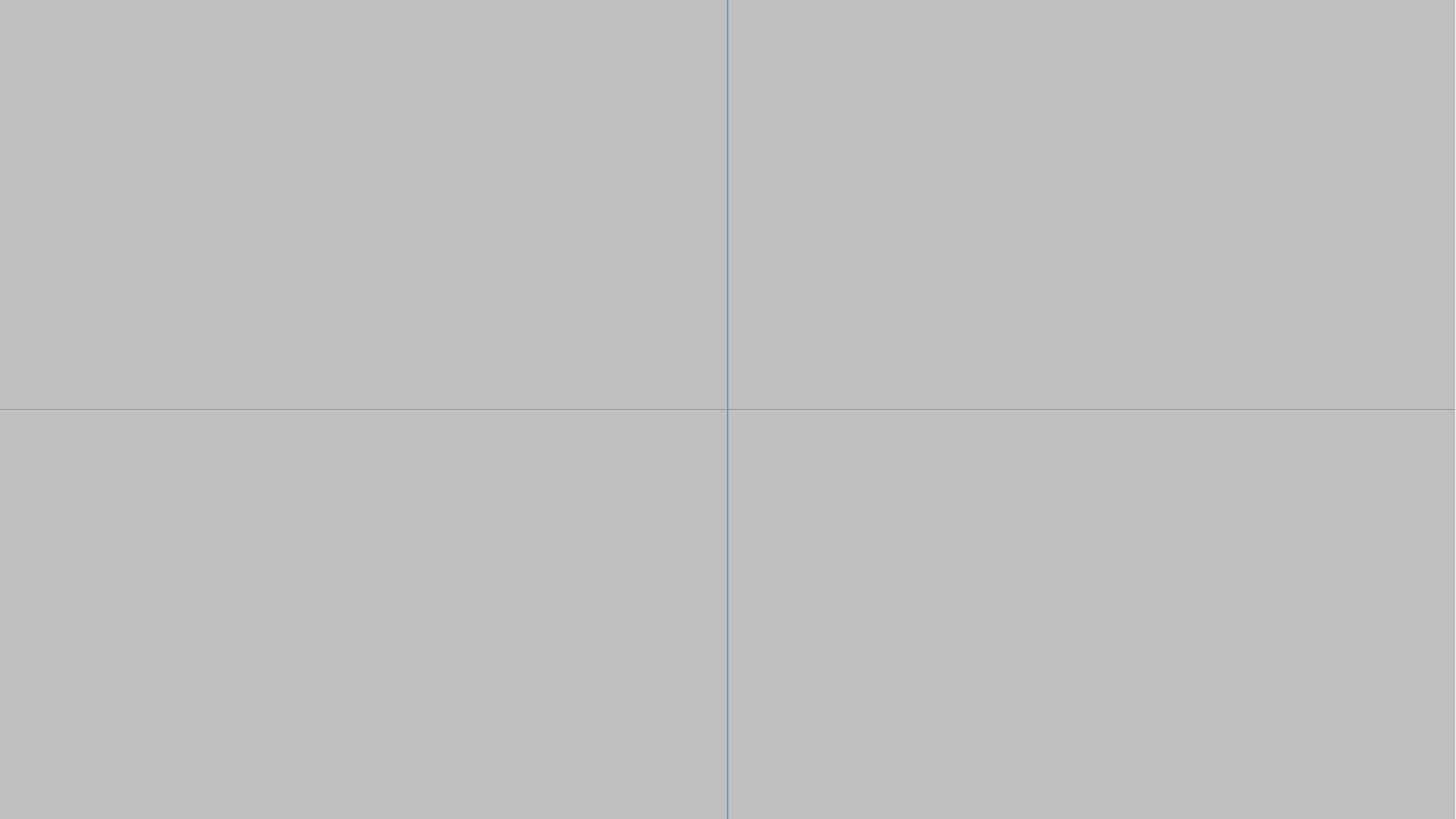

## Slide 2
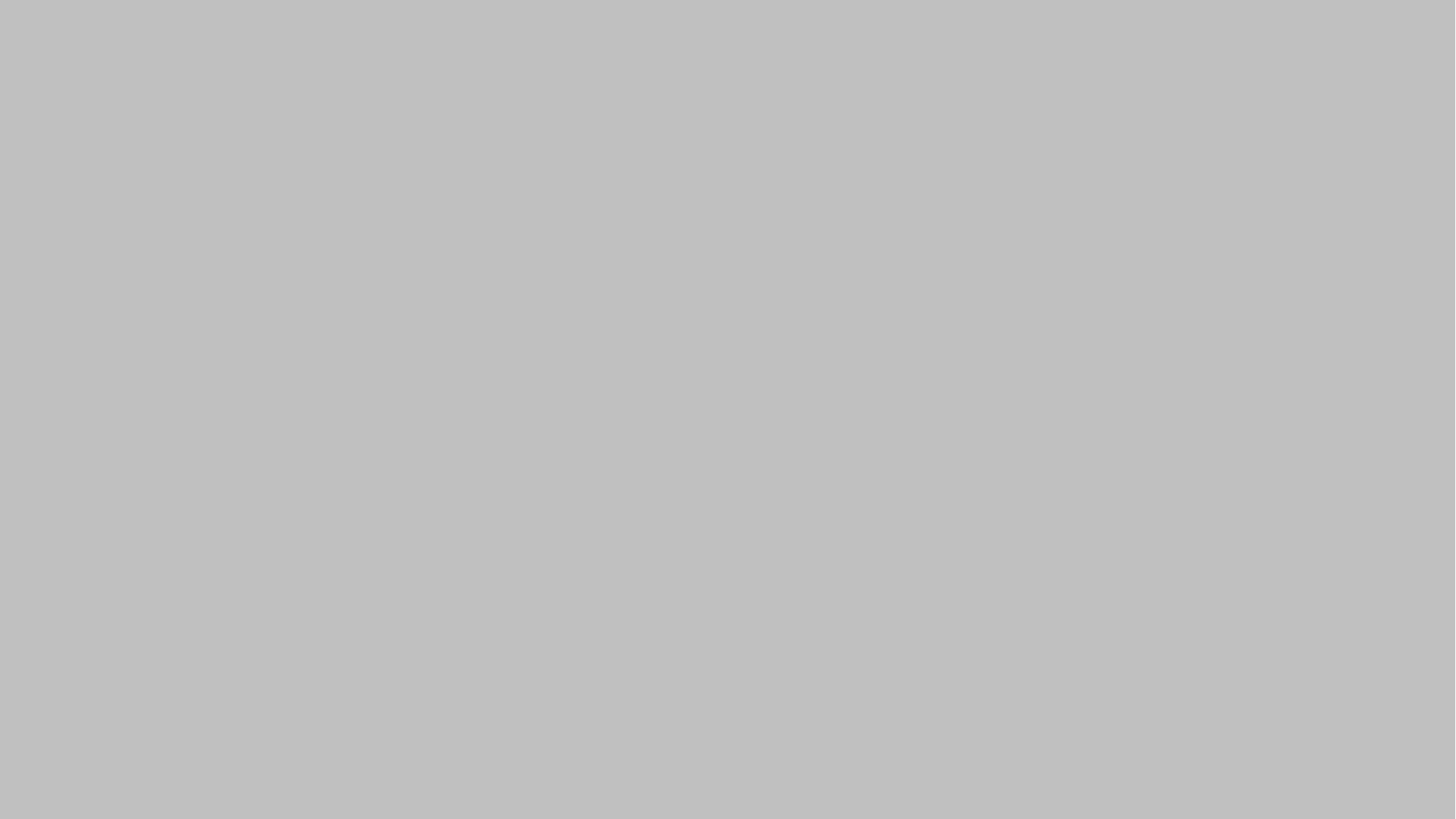

## Slide 3
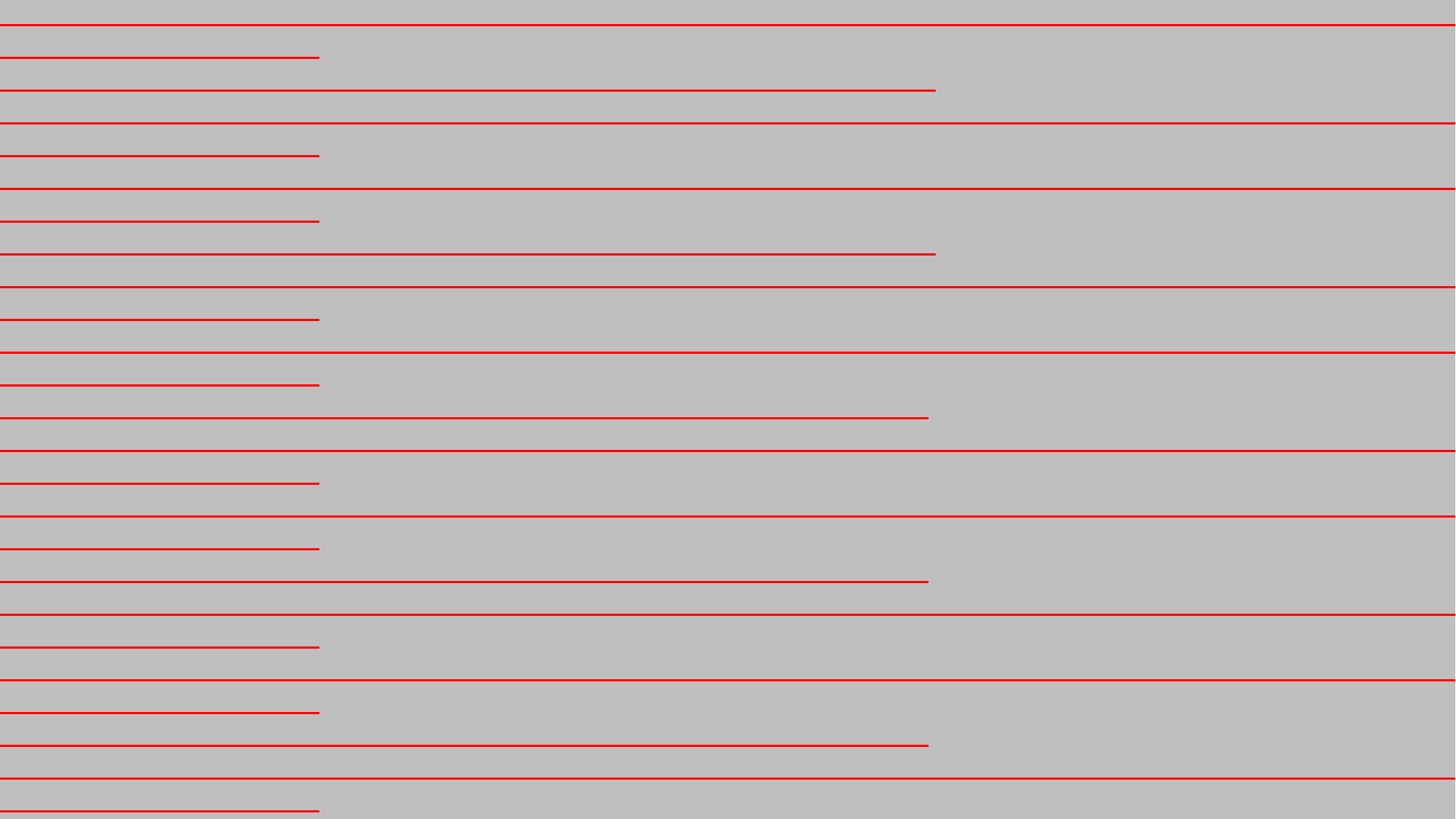

_____________________________________________________________________________________
__________________________________________________________________________________________________________________________________________________________________________
__________________________________________________________________________________________________________________________________________________________________________
_____________________________________________________________________________________
__________________________________________________________________________________________________________________________________________________________________________
__________________________________________________________________________________________________________________________________________________________________________
_____________________________________________________________________________________
__________________________________________________________________________________________________________________________________________________________________________
__________________________________________________________________________________________________________________________________________________________________________
_____________________________________________________________________________________
__________________________________________________________________________________________________________________________________________________________________________
__________________________________________________________________________________________________________________________________________________________________________
_____________________________________________________________________________________
__________________________________________________________________________________________________________________________________________________________________________
__________________________________________________________________________________________________________________________________________________________________________
_____________________________________________________________________________________
__________________________________________________________________________________________________________________________________________________________________________
__________________________________________________________________________________________________________________________________________________________________________

## Slide 4
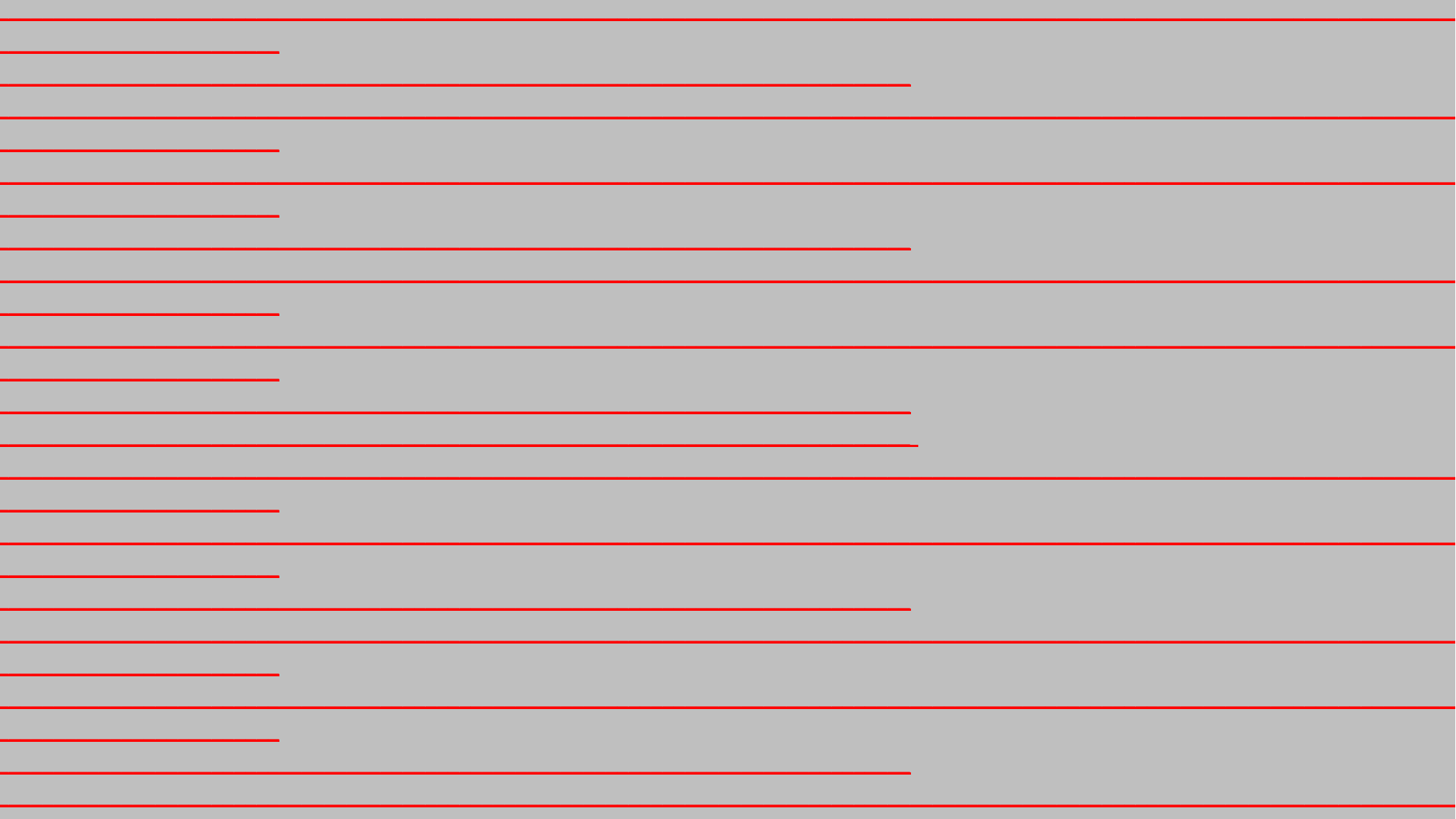

_______________________________________________________________________________________
______________________________________________________________________________________________________________________________________________________________________________
______________________________________________________________________________________________________________________________________________________________________________
_______________________________________________________________________________________
______________________________________________________________________________________________________________________________________________________________________________
______________________________________________________________________________________________________________________________________________________________________________
_______________________________________________________________________________________
______________________________________________________________________________________________________________________________________________________________________________
______________________________________________________________________________________________________________________________________________________________________________
_______________________________________________________________________________________
_______________________________________________________________________________________
______________________________________________________________________________________________________________________________________________________________________________
______________________________________________________________________________________________________________________________________________________________________________
_______________________________________________________________________________________
______________________________________________________________________________________________________________________________________________________________________________
______________________________________________________________________________________________________________________________________________________________________________
_______________________________________________________________________________________
______________________________________________________________________________________________________________________________________________________________________________
______________________________________________________________________________________________________________________________________________________________________________
_______________________________________________________________________________________
______________________________________________________________________________________________________________________________________________________________________________

## Slide 5
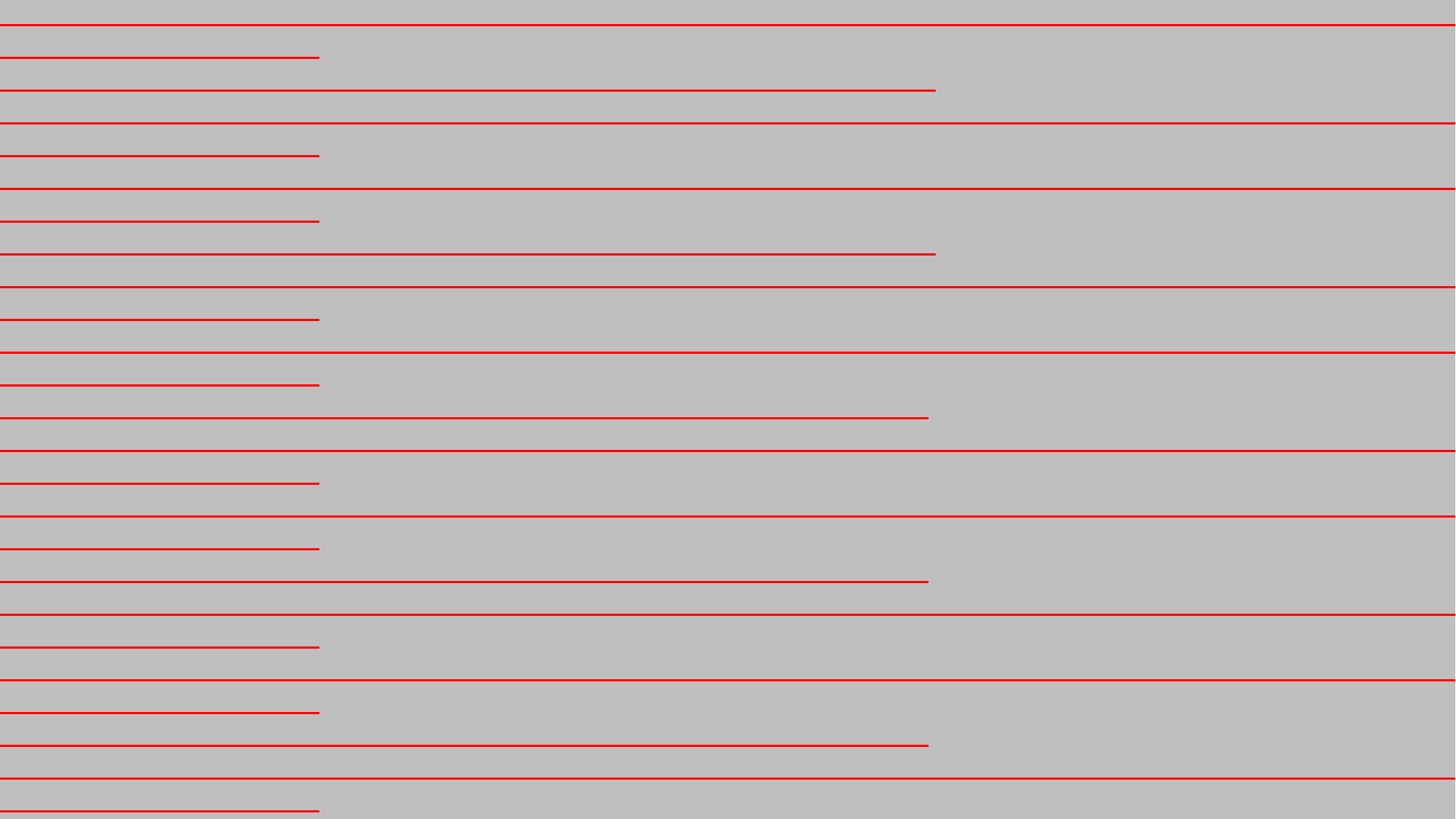

_____________________________________________________________________________________
__________________________________________________________________________________________________________________________________________________________________________
__________________________________________________________________________________________________________________________________________________________________________
_____________________________________________________________________________________
__________________________________________________________________________________________________________________________________________________________________________
__________________________________________________________________________________________________________________________________________________________________________
_____________________________________________________________________________________
__________________________________________________________________________________________________________________________________________________________________________
__________________________________________________________________________________________________________________________________________________________________________
_____________________________________________________________________________________
__________________________________________________________________________________________________________________________________________________________________________
__________________________________________________________________________________________________________________________________________________________________________
_____________________________________________________________________________________
__________________________________________________________________________________________________________________________________________________________________________
__________________________________________________________________________________________________________________________________________________________________________
_____________________________________________________________________________________
__________________________________________________________________________________________________________________________________________________________________________
__________________________________________________________________________________________________________________________________________________________________________

## Slide 6
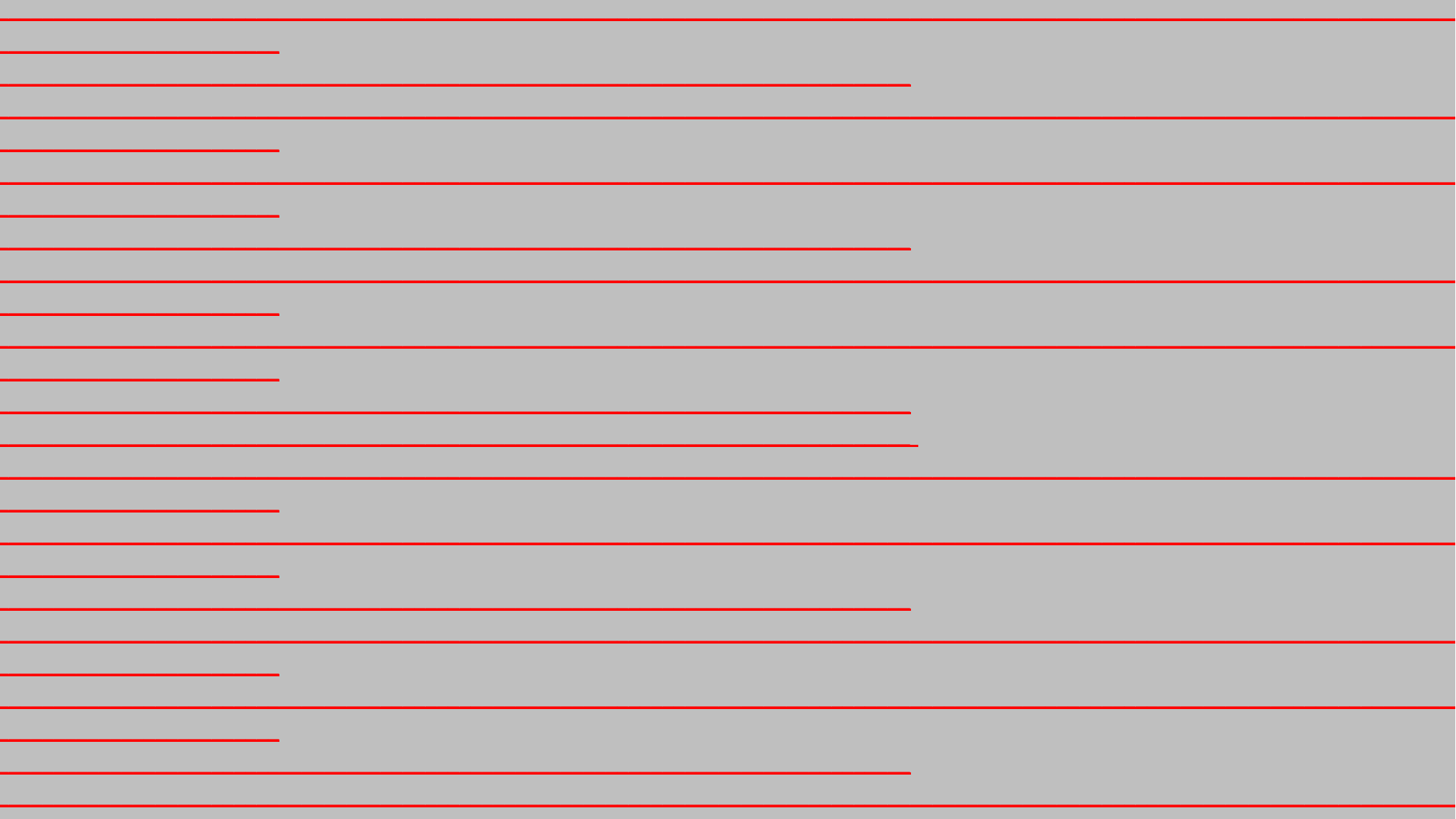

_______________________________________________________________________________________
______________________________________________________________________________________________________________________________________________________________________________
______________________________________________________________________________________________________________________________________________________________________________
_______________________________________________________________________________________
______________________________________________________________________________________________________________________________________________________________________________
______________________________________________________________________________________________________________________________________________________________________________
_______________________________________________________________________________________
______________________________________________________________________________________________________________________________________________________________________________
______________________________________________________________________________________________________________________________________________________________________________
_______________________________________________________________________________________
_______________________________________________________________________________________
______________________________________________________________________________________________________________________________________________________________________________
______________________________________________________________________________________________________________________________________________________________________________
_______________________________________________________________________________________
______________________________________________________________________________________________________________________________________________________________________________
______________________________________________________________________________________________________________________________________________________________________________
_______________________________________________________________________________________
______________________________________________________________________________________________________________________________________________________________________________
______________________________________________________________________________________________________________________________________________________________________________
_______________________________________________________________________________________
______________________________________________________________________________________________________________________________________________________________________________

## Slide 7
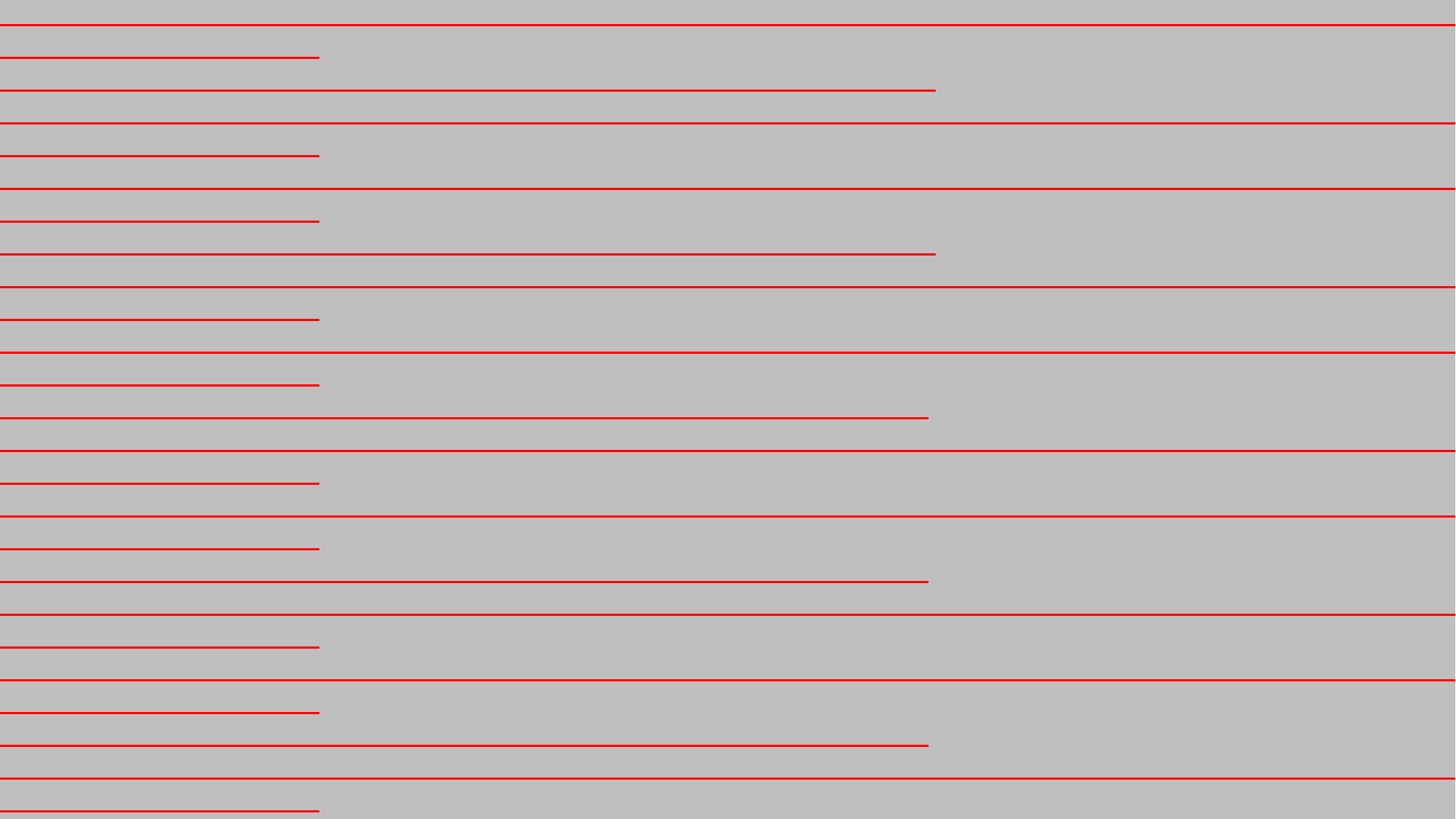

_____________________________________________________________________________________
__________________________________________________________________________________________________________________________________________________________________________
__________________________________________________________________________________________________________________________________________________________________________
_____________________________________________________________________________________
__________________________________________________________________________________________________________________________________________________________________________
__________________________________________________________________________________________________________________________________________________________________________
_____________________________________________________________________________________
__________________________________________________________________________________________________________________________________________________________________________
__________________________________________________________________________________________________________________________________________________________________________
_____________________________________________________________________________________
__________________________________________________________________________________________________________________________________________________________________________
__________________________________________________________________________________________________________________________________________________________________________
_____________________________________________________________________________________
__________________________________________________________________________________________________________________________________________________________________________
__________________________________________________________________________________________________________________________________________________________________________
_____________________________________________________________________________________
__________________________________________________________________________________________________________________________________________________________________________
__________________________________________________________________________________________________________________________________________________________________________

## Slide 8
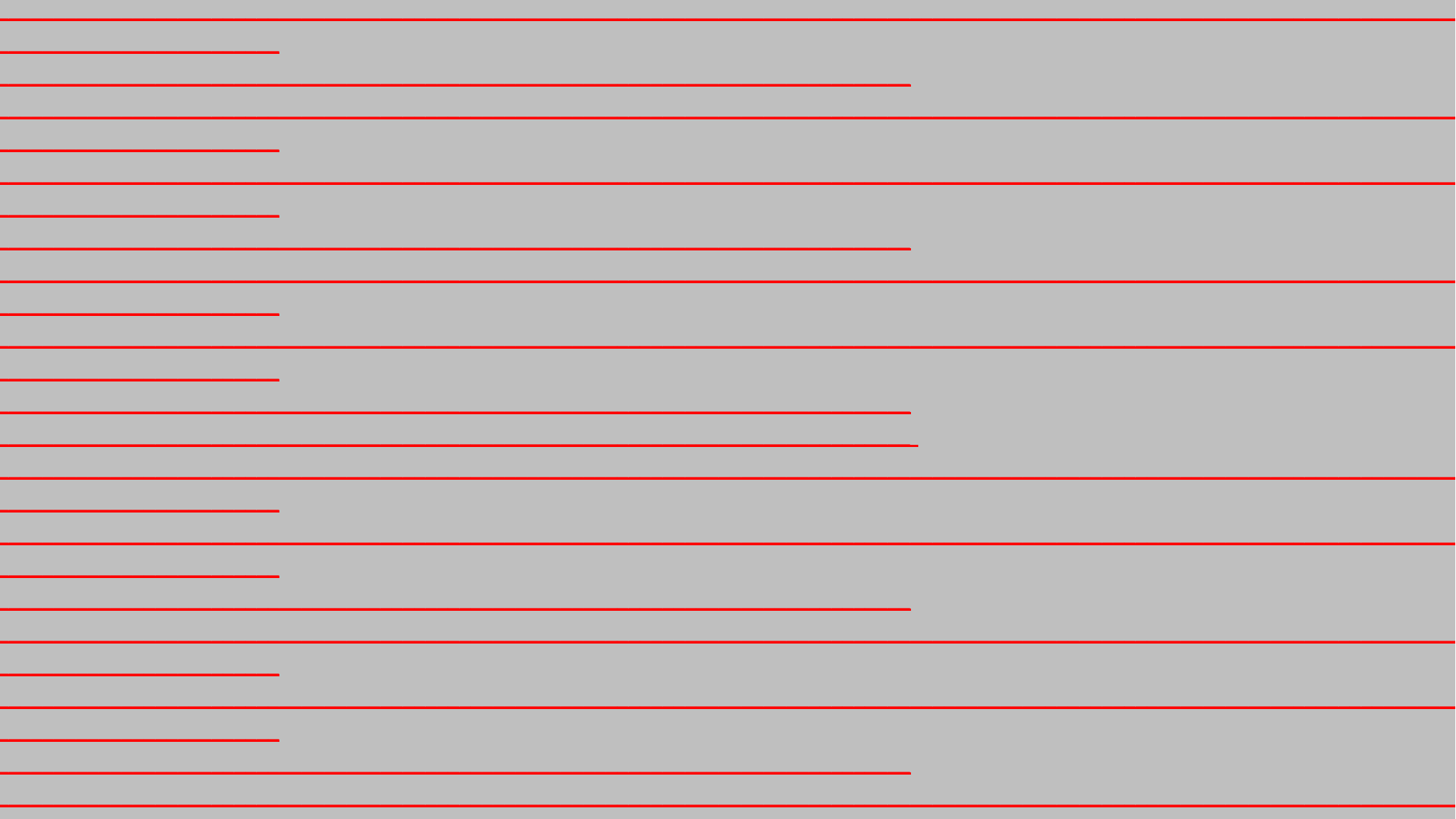

_______________________________________________________________________________________
______________________________________________________________________________________________________________________________________________________________________________
______________________________________________________________________________________________________________________________________________________________________________
_______________________________________________________________________________________
______________________________________________________________________________________________________________________________________________________________________________
______________________________________________________________________________________________________________________________________________________________________________
_______________________________________________________________________________________
______________________________________________________________________________________________________________________________________________________________________________
______________________________________________________________________________________________________________________________________________________________________________
_______________________________________________________________________________________
_______________________________________________________________________________________
______________________________________________________________________________________________________________________________________________________________________________
______________________________________________________________________________________________________________________________________________________________________________
_______________________________________________________________________________________
______________________________________________________________________________________________________________________________________________________________________________
______________________________________________________________________________________________________________________________________________________________________________
_______________________________________________________________________________________
______________________________________________________________________________________________________________________________________________________________________________
______________________________________________________________________________________________________________________________________________________________________________
_______________________________________________________________________________________
______________________________________________________________________________________________________________________________________________________________________________

## Slide 9
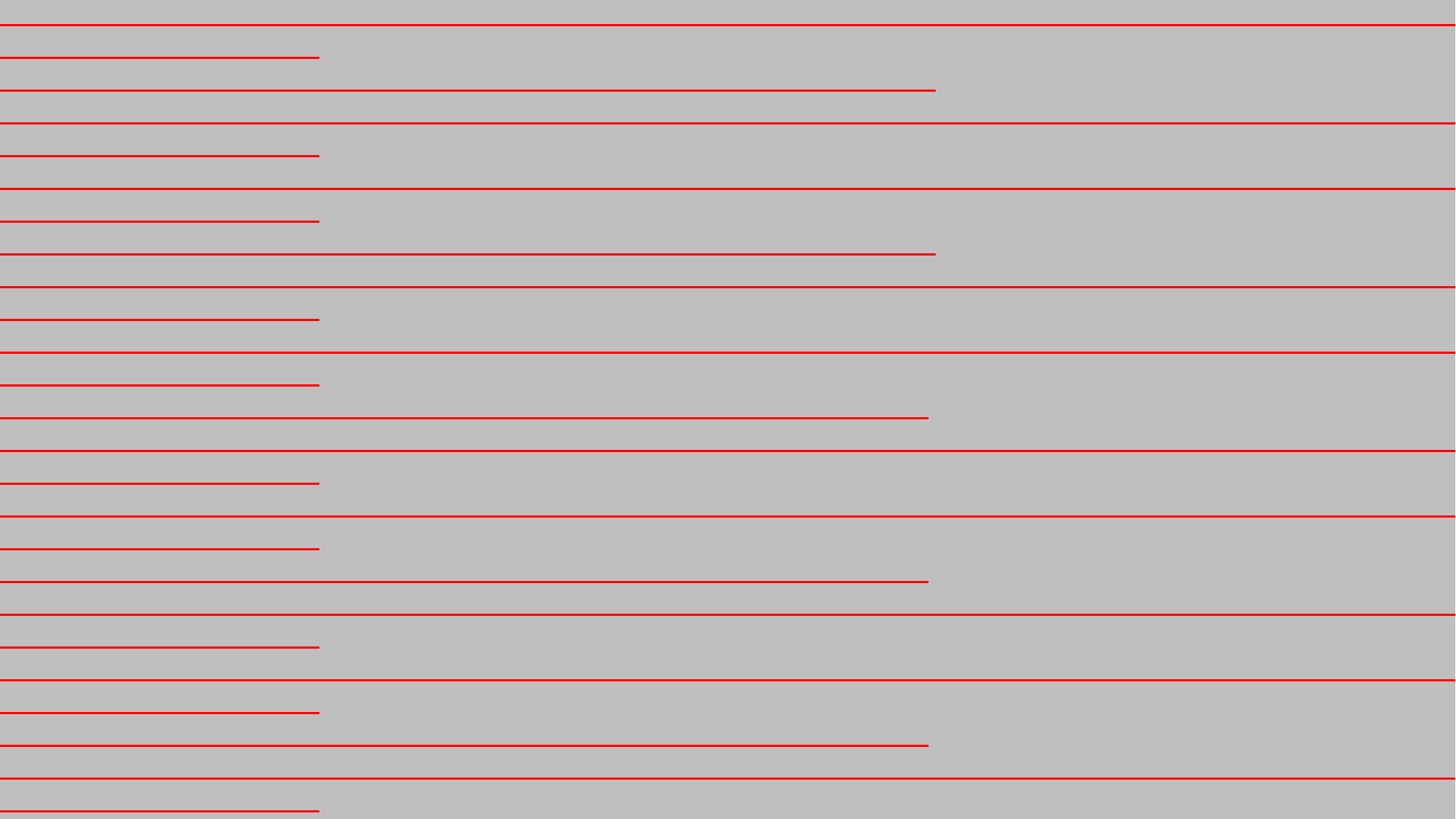

_____________________________________________________________________________________
__________________________________________________________________________________________________________________________________________________________________________
__________________________________________________________________________________________________________________________________________________________________________
_____________________________________________________________________________________
__________________________________________________________________________________________________________________________________________________________________________
__________________________________________________________________________________________________________________________________________________________________________
_____________________________________________________________________________________
__________________________________________________________________________________________________________________________________________________________________________
__________________________________________________________________________________________________________________________________________________________________________
_____________________________________________________________________________________
__________________________________________________________________________________________________________________________________________________________________________
__________________________________________________________________________________________________________________________________________________________________________
_____________________________________________________________________________________
__________________________________________________________________________________________________________________________________________________________________________
__________________________________________________________________________________________________________________________________________________________________________
_____________________________________________________________________________________
__________________________________________________________________________________________________________________________________________________________________________
__________________________________________________________________________________________________________________________________________________________________________

## Slide 10
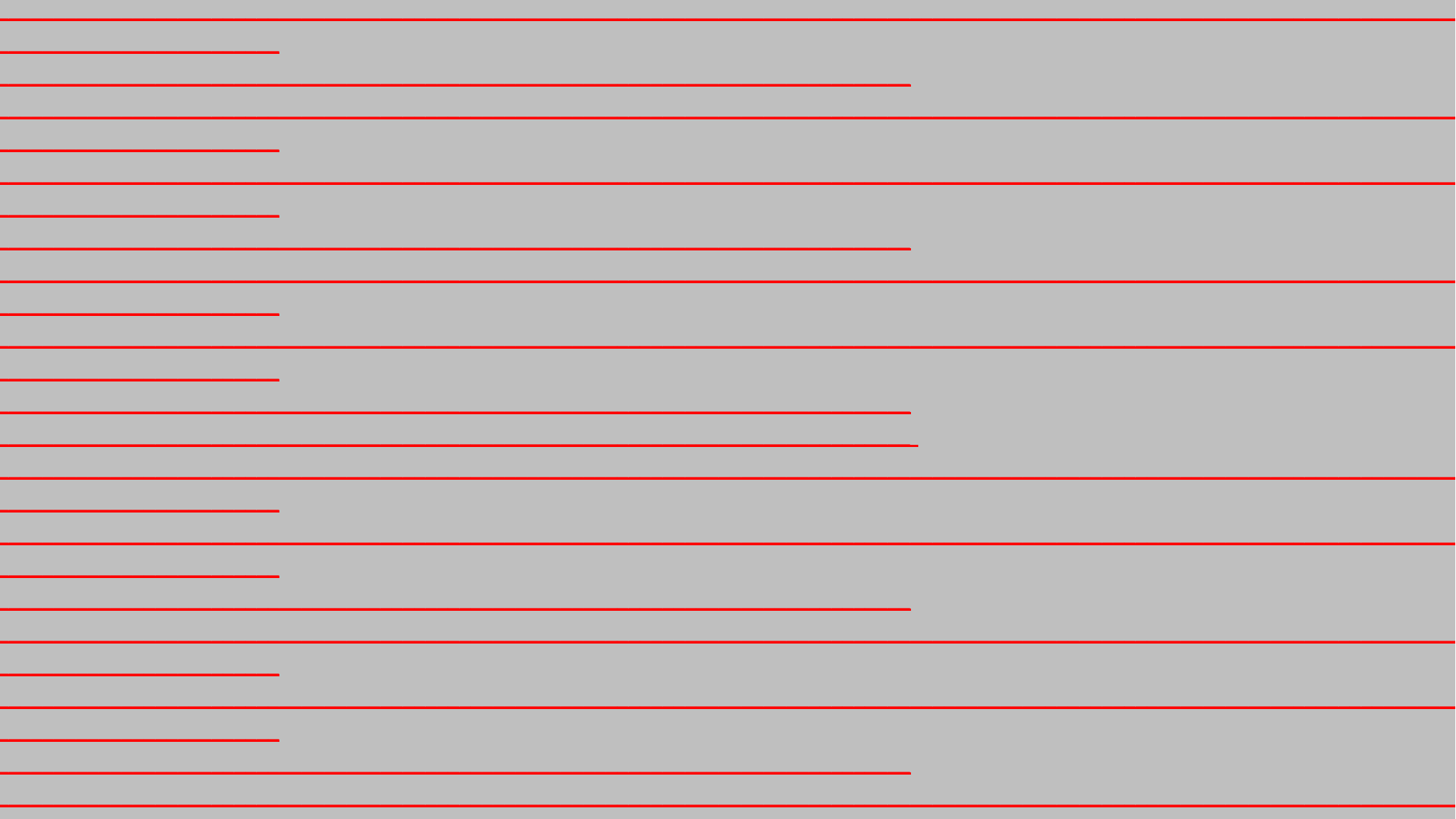

_______________________________________________________________________________________
______________________________________________________________________________________________________________________________________________________________________________
______________________________________________________________________________________________________________________________________________________________________________
_______________________________________________________________________________________
______________________________________________________________________________________________________________________________________________________________________________
______________________________________________________________________________________________________________________________________________________________________________
_______________________________________________________________________________________
______________________________________________________________________________________________________________________________________________________________________________
______________________________________________________________________________________________________________________________________________________________________________
_______________________________________________________________________________________
_______________________________________________________________________________________
______________________________________________________________________________________________________________________________________________________________________________
______________________________________________________________________________________________________________________________________________________________________________
_______________________________________________________________________________________
______________________________________________________________________________________________________________________________________________________________________________
______________________________________________________________________________________________________________________________________________________________________________
_______________________________________________________________________________________
______________________________________________________________________________________________________________________________________________________________________________
______________________________________________________________________________________________________________________________________________________________________________
_______________________________________________________________________________________
______________________________________________________________________________________________________________________________________________________________________________

## Slide 11
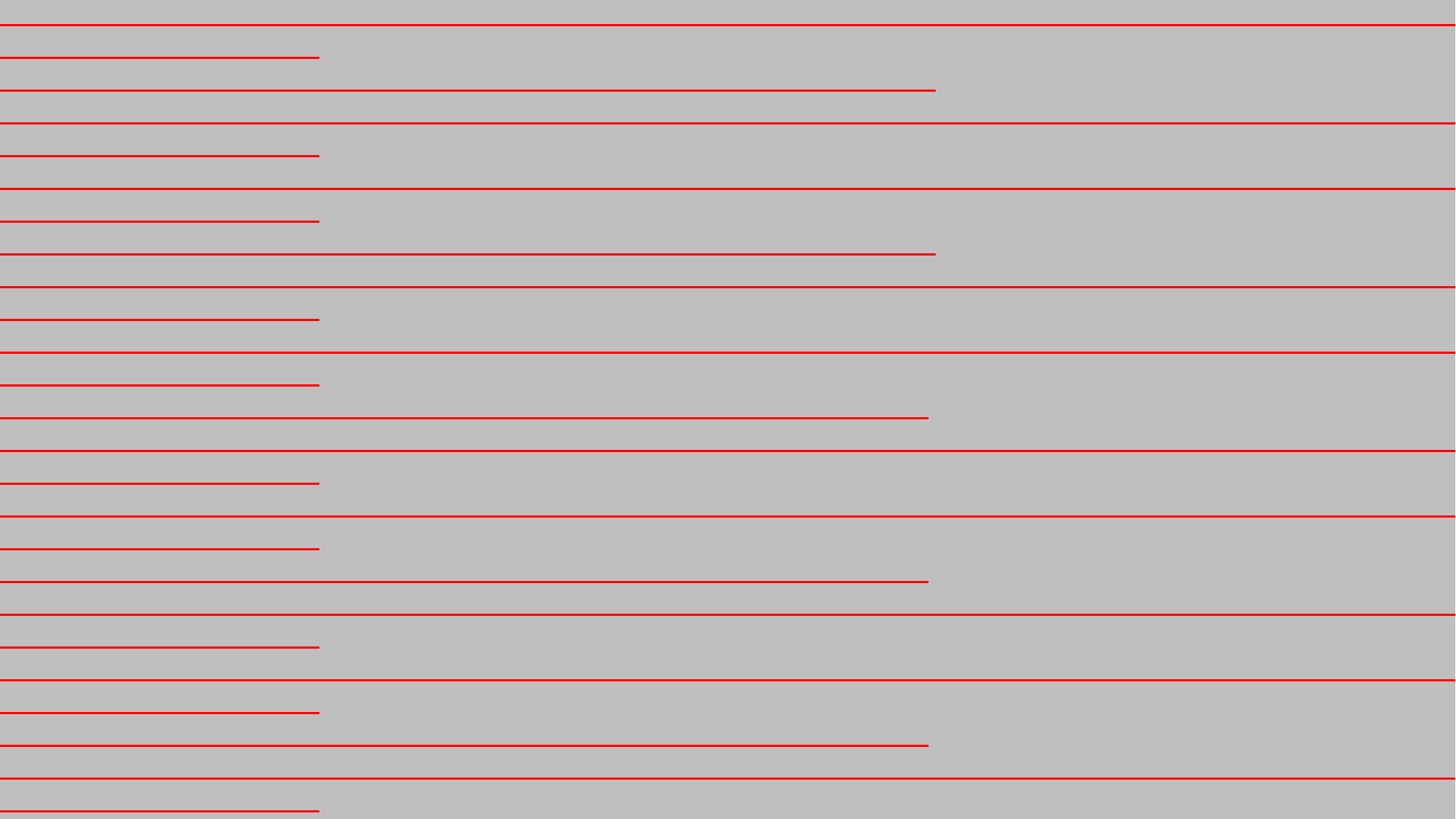

_____________________________________________________________________________________
__________________________________________________________________________________________________________________________________________________________________________
__________________________________________________________________________________________________________________________________________________________________________
_____________________________________________________________________________________
__________________________________________________________________________________________________________________________________________________________________________
__________________________________________________________________________________________________________________________________________________________________________
_____________________________________________________________________________________
__________________________________________________________________________________________________________________________________________________________________________
__________________________________________________________________________________________________________________________________________________________________________
_____________________________________________________________________________________
__________________________________________________________________________________________________________________________________________________________________________
__________________________________________________________________________________________________________________________________________________________________________
_____________________________________________________________________________________
__________________________________________________________________________________________________________________________________________________________________________
__________________________________________________________________________________________________________________________________________________________________________
_____________________________________________________________________________________
__________________________________________________________________________________________________________________________________________________________________________
__________________________________________________________________________________________________________________________________________________________________________

## Slide 12
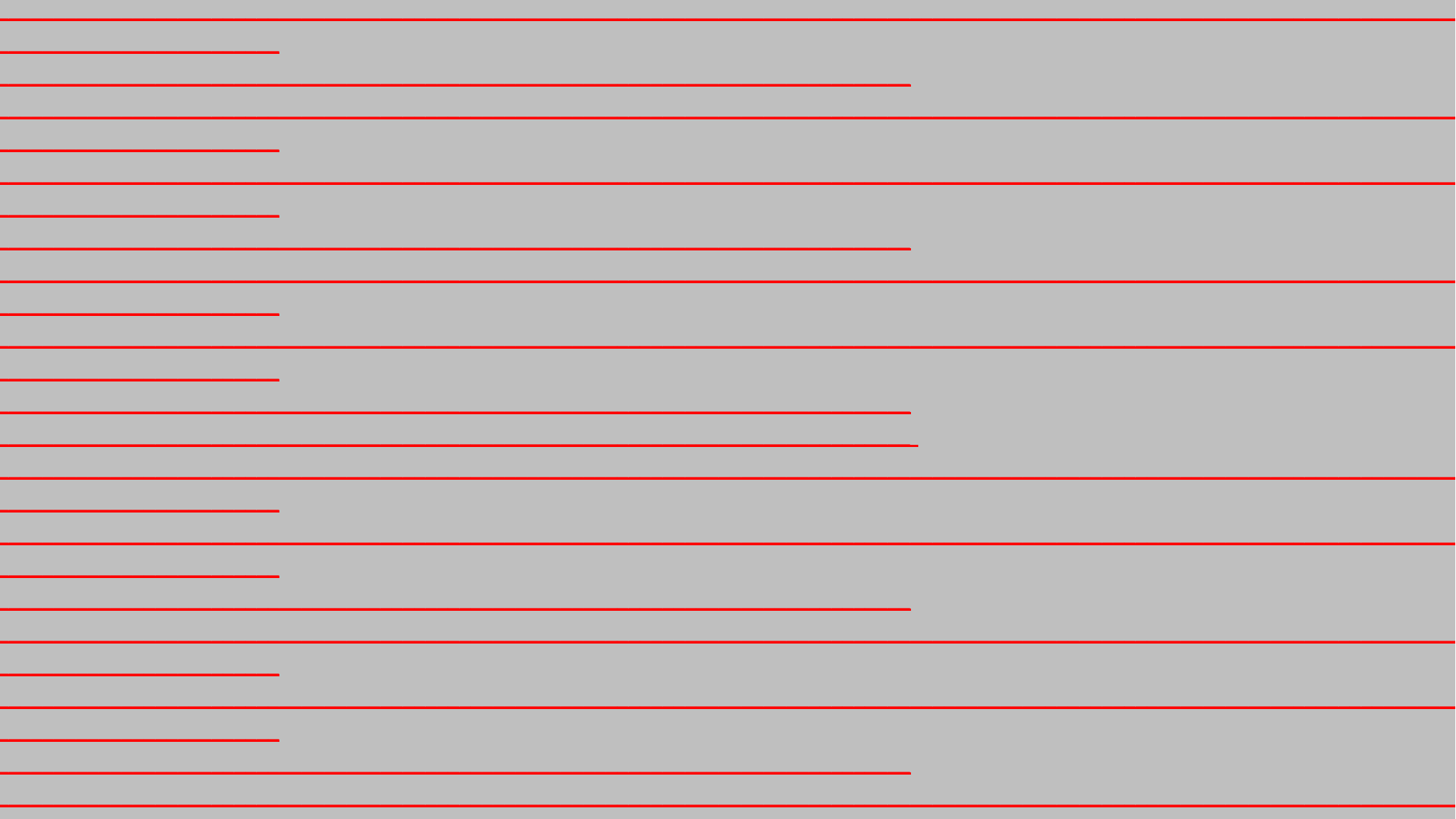

_______________________________________________________________________________________
______________________________________________________________________________________________________________________________________________________________________________
______________________________________________________________________________________________________________________________________________________________________________
_______________________________________________________________________________________
______________________________________________________________________________________________________________________________________________________________________________
______________________________________________________________________________________________________________________________________________________________________________
_______________________________________________________________________________________
______________________________________________________________________________________________________________________________________________________________________________
______________________________________________________________________________________________________________________________________________________________________________
_______________________________________________________________________________________
_______________________________________________________________________________________
______________________________________________________________________________________________________________________________________________________________________________
______________________________________________________________________________________________________________________________________________________________________________
_______________________________________________________________________________________
______________________________________________________________________________________________________________________________________________________________________________
______________________________________________________________________________________________________________________________________________________________________________
_______________________________________________________________________________________
______________________________________________________________________________________________________________________________________________________________________________
______________________________________________________________________________________________________________________________________________________________________________
_______________________________________________________________________________________
______________________________________________________________________________________________________________________________________________________________________________

Supplement: Supplementary file 3 — Supplement 3 [file 41598_2019_50372_MOESM3_ESM.zip › S3_PowerPoint_80_min_blank_100_min_red_lines.pptx]
