# Supplementary material for: Analysis of vertebrate vision in a 384-well imaging system
Source: Sci Rep. 2019 Sep 27;9:13989. doi: 10.1038/s41598-019-50372-0 (PMC6764987; doi:10.1038/s41598-019-50372-0)
Supplement: Supplementary file 4 — Supplement 4 [file 41598_2019_50372_MOESM4_ESM.pdf]

Analysis of vertebrate vision in a 384-well imaging system

Robert J. Thorn[1], Amanda Dombroski[2], Kerry Eller[1], Tania M. Dominguez[1], Danielle E. Clift[1], Peter Baek[1], Renee J. Seto[1], Elizabeth S. Kahn[1], Sara K. Tucker[1], Ruth M. Colwill[3], Jason K. Sello[2], Robbert Creton[1]

1. Department of Molecular Biology, Cell Biology and Biochemistry, 2. Department of Chemistry, 3. Department of Cognitive, Linguistic and Psychological Sciences, Brown University, Providence, RI 02912, USA

Supplement 4: Equipment and source list

1) Cabinet: any tall cabinet measuring at least 180 × 40 × 40 cm
2) Canon EOS Rebel T6 digital camera with an EF-S 55–250 mm f/4.0–5.6 IS zoom lens
3) Canon Remote Capture software (EOS Utility, version 3, included with the camera)
4) Canon camera power supply (Canon ACK-E10 AC Adapter)
5) Two USB cables to connect the laptop to the camera and projector
6) M5 LED pico projector (Aaxa Technologies)
7) Laptop or desktop computer (PC); e.g. Acer Aspire or Dell Latitude 5490
8) MS Office with PowerPoint
9) Bessey 4.5 inch bench vise
10) Heating pad 15 x 12 inches (Millard)
11) Temperature control unit (GeekTeches, TMC-1000)
12) Shelf brackets (Rubbermaid 25 in. Black Twin Track & 11.5 in. Black Twin Track Bracket)
13) Glass plate (Home Depot, model 91114, 11 x 14 inches).
14) Diffuser (ePlastics, 2447 white acrylic sheet, 0.060 inch thick, 11 x 7 inch panel)
15) White 96-well ProxiPlates (PerkinElmer, 6006290)
16) Work station (PC) with MS Excel for image analysis
17) ImageJ, which can be downloaded free of charge from the NIH
18) ImageJ macro for automated image analysis (supplement 1)
19) MS Excel template for data analysis and visualization (supplement 2)
20) PowerPoint presentation with visual stimuli (supplement 3)
